# Fibroadenoma in a transgender woman

**DOI:** 10.1093/jscr/rjad202

**Published:** 2023-04-20

**Authors:** Andrew S Beatty, Diana Tam

**Affiliations:** Department of General Surgery, The Prince Charles Hospital, Brisbane, QLD, Australia; Northside Clinical School, School of Medicine, The University of Queensland, Brisbane, QLD, Australia; Department of General Surgery, The Prince Charles Hospital, Brisbane, QLD, Australia; Department of General Surgery, The Royal Brisbane Women’s Hospital Brisbane, Brisbane, QLD, Australia

## Abstract

A breast fibroadenoma is a common benign condition that is typically seen in younger women. Rarely has it been described in men and often because of prescribed medications, but for transgender women, this occurrence is even rarer. Endocrine therapy plays a key role in the transition of a transwoman from male to female of which oestrogen is the most common form. The use of cross-sex hormones such as oestrogen is becoming increasingly more utilized in this transition helping in feminization but also secondary breast development. However, this does result in the development of other breast pathologies that were almost never seen in their *cis*-male counterparts. Herein, we present the case of a 27-year-old transwoman who presented with a palpable breast lump after being on oral oestrogen therapy for 6 years. She proceeded to an excisional biopsy, which confirmed the lesion to be a benign fibroadenoma.

## INTRODUCTION

Fibroadenomas of the breast are a common benign condition typically seen in younger woman, but for transgender women, this condition is incredibly rare. Since being first described in the mid-nineteenth century, the transgender population has undergone massive expansion with international studies demonstrating increasing referrals to gender clinics to assist with gender transition [1]. With this population growth, we are now starting to recognize their unique healthcare needs and how conditions in transgender individuals can present differently from their cis counterparts. Herein, we present the case of a 27-year-old transgender woman (male-to-female) who presented with a palpable breast lesion that underwent an excisional biopsy, which confirmed the lesion to be a fibroadenoma.

## CASE REPORT

A 27-year-old transgender female was seen at the surgical outpatient clinic with a self-detected right breast lump present for 12 months. The lump became apparent following a period of intentional weight loss of ~5 kg. There are no associated overlying skin changes, dimpling, pain nor nipple discharge. She had no recollection of trauma to the breast. She had a past medical history of depression and began gender transition from male to female at the age of 21. She was commenced on oral oestrogen as her endocrine therapy and the dosage had been stable for 3 years. She had not undergone any gender affirming surgery or prior breast augmentation. She had a family history of breast cancer with a maternal aunt being diagnosed in her mid-30s.

**Figure 1:**
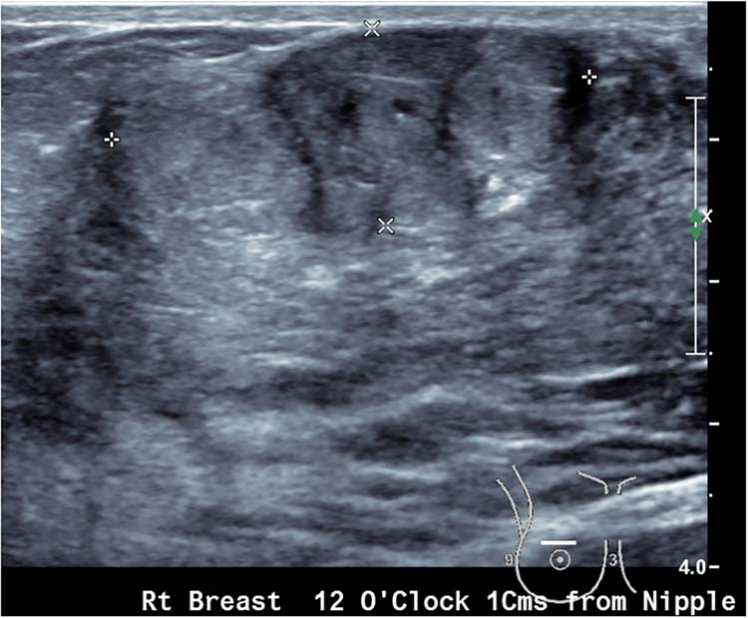
Ultrasound image showing a lobulated ovoid breast lesion.

**Figure 2:**
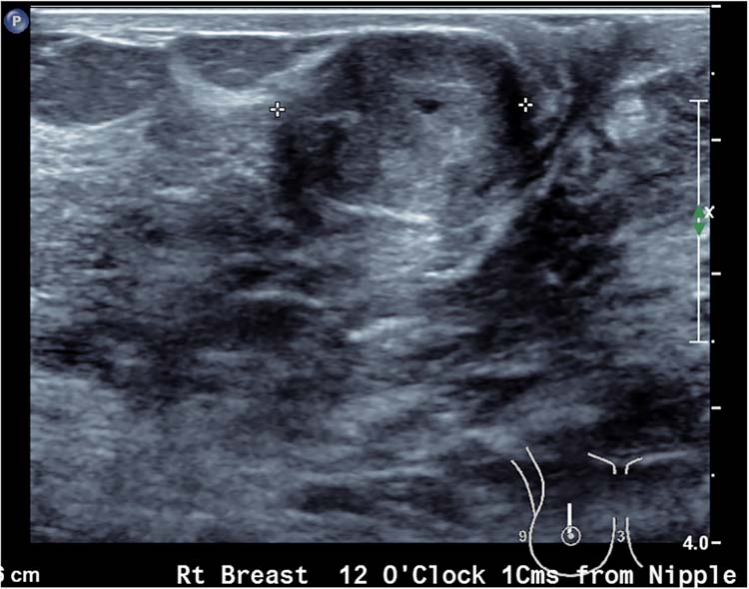
Ultrasound image of the ovoid breast lesion.

Upon examination, she has B cup breasts. A firm mobile 3 cm lump was palpated at 10 o’clock deep to the areolar edge and there was no palpable axillary lymphadenopathy. The contralateral breast was unremarkable. Ultrasound assessment of the breast demonstrated a lobulated lesion with minimal calcifications measuring 31 × 14 × 19 mm (Figs 1 and 2). No other lesions were detected, and no suspicious lymph nodes were present within the ipsilateral axilla. She proceeded to a core biopsy which demonstrated a fibroepithelial lesion with low cellularity and mitotic figures. A low grade phyllodes tumour could not be excluded based on the core biopsy. Following the discussion of ongoing surveillance, repeat core biopsy or excisional biopsy, the patient decided to proceed with an excision of the lesion.

The lesion was successfully excised without issue. Histopathology of the lesion demonstrated a biphasic growth pattern comprised of an epithelial and stromal component. There was no *in situ* or invasive malignancy consistent with the lesion being a benign fibroadenoma. She was reviewed in the outpatient clinic with her wound fully healed and has had no further issues.

## DISCUSSION

To our knowledge, there have only been three other documented cases of fibroadenoma in transgender individuals [2–4]. If we compare against *cis*-men, a fibroadenoma was at a time considered to be impossible in men. Indeed, Holleb *et al*. [5] concluded that there was no true fibroadenoma within a *cis*-male. Shin and Rosen [6, 7] proposed the pathophysiology of a fibroadenoma in a man to be part of the disease process of gynaecomastia given they could not find any cases of fibroadenoma without gynaecomastia. In such reported cases, there appears to be an association with prescribed medications including antiandrogen therapy [4].

It has been established that fibroadenomas can possess both oestrogen and progesterone receptors hence the cross-sex hormones prescribed to our patient were the likely influence for the development of this lesion [8]. It has also been reported under the influence of cross-sex hormones that the *cis*-male breast becomes histologically indistinguishable from the *cis*-female breast tissue [9]. If indeed the oestrogen therapy contributed to the development of the fibroadenoma in this case, given the increasing use of endocrine therapy for gender reassignment this may become an increasingly more commonly seen pathology of the breast in transgender women.

Breast development because of exogenous oestrogen appears to follow muted Tanner stages similar to pubescent development but the degree of development appears to be independent of type and dose of hormonal therapy [10]. Given the lobule formation is similar to that of natal women the appearance of any lesions with imaging is identical between transgender and *cis*-women [11].

Unfortunately, not all lesions of the breast in transgender women are benign. Symmers [12] described the first case of breast cancer in a transgender woman and since then there have been several reported cases in both transwomen and transmen [13, 14]. While the exact risk to this population is unclear, the repeated cases do highlight that malignancy is a differential that must be considered and excluded. However, over testing to exclude malignancy is not required rather the same index of suspicion should be applied to transwomen as compared with *cis*-women as their ratio of benign to malignant lesions is comparable to that of *cis*-women [15].

## CONCLUSION

Just as a fibroadenoma in a *cis*-woman is a benign condition that carries no malignant potential, there is no evidence to suggest in a transgender individual that it should be any different. However, breast cancer in this cohort has been repeatedly demonstrated and as such any new lesion within a breast should always have malignancy excluded regardless of age or gender at birth.
